# Hidden blood loss in adolescent idiopathic scoliosis patients undergoing posterior spinal fusion surgery: a retrospective study of 765 cases at a single centre

**DOI:** 10.1186/s12891-021-04681-z

**Published:** 2021-09-15

**Authors:** Lipeng Wang, Jiangli Liu, Xiaoxiao Song, Muhui Luo, Yongquan Chen

**Affiliations:** 1 Department of Anaesthesia, Yijishan Hospital of Wannan Medical College, No. 2, Zheshan West Road, Wuhu, Anhui China; 2 Department of Orthopedics, Affiliated Taikang Xianlin Drum Tower Hospital, Medical school of Nanjing University, Nanjing, Jiangsu China; 3 Department of Anaesthesia, The First Affiliated Hospital of Nanchang University, Nanchang, Jiangxi China

**Keywords:** Hidden blood loss (HBL), Adolescent idiopathic scoliosis, Risk factors, Multivariate linear regression analysis, Posterior spinal fusion surgery, Postoperative blood transfusion

## Abstract

**Background:**

In scoliosis corrective surgery, total blood loss is composed of visible blood loss, including intraoperative haemorrhage and drainage, and hidden blood loss in which blood extravasates into the tissues and accumulates in the surgical field. The purpose of this study was to investigate hidden blood loss (HBL) and its potential risk factors in adolescent idiopathic scoliosis patients undergoing posterior spinal fusion surgery and elucidate the influence of HBL on the necessity for postoperative blood transfusion.

**Methods:**

We retrospectively studied adolescent idiopathic scoliosis patients undergoing posterior spine fusion for adolescent idiopathic scoliosis from January 2014 to December 2018 at our hospital. The patients’ demographics, blood loss-related parameters, surgeries and blood loss data were extracted. The association between patient characteristics and HBL was analyzed by Pearson or Spearman correlation analyses. Multivariate linear regression analysis was used to determine independent risk factors associated with HBL. Binary logistic regression analysis was used to analyze the influence of HBL on the necessity for postoperative blood transfusion.

**Results:**

A total of 765 patients, of whom 128 were male and 637 were female (age range 10–18 years), were included in this study. The mean volume of HBL was 693.5 ± 473.4 ml, accounting for 53.9 % of the total blood loss. The multivariate linear regression analysis revealed that preoperative Hct (*p* = 0.003) and allogeneic blood transfusion (*p* < 0.001) were independent risk factors for HBL, while tranexamic acid (*p* = 0.003) was negatively correlated with HBL. Binary logistic regression analysis showed that HBL > 850 ml (*P* < 0.001, OR: 8.845, 95 % CI: 5.806–13.290) was an independent risk factor for the necessity for postoperative blood transfusion.

**Conclusions:**

Substantial HBL occurred in adolescent idiopathic scoliosis patients undergoing posterior spinal fusion surgeries. Allogeneic blood transfusion and preoperative Hct were independent risk factors for HBL, while tranexamic acid was negatively related to HBL. HBL and its influencing factors should be considered when planning perioperative transfusion management. Patients with HBL greater than 850 ml should be closely monitored in cases of postoperative anaemia.

**Level of evidence:**

Level III.

## Background

Blood loss remains a major focus in orthopaedic surgery for adolescent idiopathic scoliosis due to extensive and complex operating procedures. Anaesthesiologists and surgeons might primarily consider visible blood loss, including postoperative drainage, and ignore the component that penetrates the tissues, residual blood in the vertebral canal and blood loss due to haemolysis, which is known as hidden blood loss [1]. The concept of HBL was first proposed by Sehat [2] and has received increasing attention from clinicians in recent years. Xu et al. [3] showed that the HBL in posterior lumbar interbody fusion surgery accounted for approximately 61 % of the total blood loss in rheumatoid arthritis patients. Kai et al. [4] also reported that the volume of HBL reached 1084 ml, accounting for 82 % of total blood loss, after hip hemiarthroplasty. HBL could exacerbate postoperative haemoglobin level reductions, resulting in increased blood transfusion requirements. Clarifying HBL will be helpful for clinicians to improve clinical assessment capabilities and manage postoperative blood transfusion. However, to date, few published studies have examined HBL in posterior spinal fusion surgeries for adolescent idiopathic scoliosis. In this study, we retrospectively explored the volume of HBL in adolescent idiopathic scoliosis surgery patients and analyzed the influencing factors of HBL.

A previous study reported that 23.4 % of patients required postoperative transfusion in posterior spinal fusion surgeries for adolescent idiopathic scoliosis [5]. Furthermore, we also found that a considerable number of patients still experienced anaemia after surgery at our medical centre, which required transfusion. Postoperative anaemia has a negative effect on illness recovery and leads to an extended hospital stay. This phenomenon may be associated with hidden blood loss. Therefore, clarifying the relationship between hidden blood loss and postoperative transfusion will guide perioperative blood transfusion practices.

## Materials and methods

### Patients

Institutional review board approval was waived as patients’ private information was not retrieved. A total of 765 patients undergoing posterior spinal fusion surgeries for adolescent idiopathic scoliosis from January 2014 to December 2018 were included in current study. All surgeries were performed by highly experienced surgeons using a uniform approach. The inclusion criteria were as follows: patients (1) younger than 18 years and diagnosed adolescent idiopathic scoliosis; (2) who underwent posterior spinal fusion surgery; and (3) obtained stable perioperative fluid balance and haemodynamics. The exclusion criteria were as follows: patients (1) with coagulation disorders or perioperative infection; (2) who were treated with anticoagulant drugs; (3) who experienced intraoperative blood loss greater than 2.5 L on account of larger bias with excessive blood loss [6]; and (4) who suffered cerebrospinal fluid leakage.

### Date extraction

Demographic information was collected from electronic medical records, including age, sex, height, weight, body mass index (BMI), preoperative and postoperative haematocrit (Hct) and haemoglobin (Hb), American Society of Anaesthesiologists (ASA) classification, preoperative Cobb angle, prothrombin time and activated partial thromboplatin time. Surgery information included operation time, pedicle screw number, bone mineral density, the number of surgical segments, whether osteotomy was performed, the use of tranexamic acid, intraoperative blood loss, postoperative drainage volume, and allogeneic and autologous blood transfusion volume.

### Blood loss management and calculation of HBL

Intraoperative blood loss was recorded by the anaesthesiologist and was mainly determined from the volume of blood in the suction apparatus and in the soaked gauzes that were used during the entire operation. Postoperative blood loss was calculated by measuring the blood volume in the haemovac. Most of the haemovac was removed on the third postoperative day. If the drainage tube could not be removed when calculating the blood loss, we measured the blood in the drainage tube on the day when the blood was taken. The total visible blood loss was calculated as the sum of the volume of intraoperative blood loss and the volume of postoperative drainage. Blood lost during surgery could be collected by the blood salvage technique and reinfused into the patient as autologous blood; whether to perform this procedure was decided by the anaesthesiologist. Patients were given a blood transfusion when the haemoglobin level was below 70 g/dL or below 80 g/dL with significant symptoms of anaemia, such as increased heart rate hypotension. We calculated the patient blood volume (PBV) according to the formula described by Nadler et al. [7]: PBV (L) = k1 × height (m)^3^ + k2 × weight (kg) + k3, (k1 = 0.3669, k2 = 0.03219, k3 = 0.6041 for males, and k1 = 0.3561, k2 = 0.03308, k3 = 0.1833 for females). The total red cell volume can be calculated by multiplying the PBV and the patient Hct together. Therefore, a reduction in Hct reflects a change in red cell volume [8]. As the haemorrhage continues, the transfusion is used to sustain the patient’s circulating volume. The linear formula proposed by Gross was found to intimately follow a logarithmic formula [9]. According to the Gross formula [9], total blood loss = PBV (Hct_pre_ - Hct_post_)/Hct_ave_, where Hct_pre_ is the initial preoperative Hct, Hct_post_ was Hct on the second or the third day postoperatively, and Hct_ave_ is the average of the Hct_pre_ and the Hct_post_. Hidden blood loss was calculated by subtracting the visible blood loss from the total blood loss according to the formula of Sehat et al. [10] as follows:

$$\mathrm{Hidden}\;\mathrm{blood}\;\mathrm{loss}\;=\;\mathrm{total}\;\mathrm{blood}\;\mathrm{loss}\;-\;\mathrm{visible}\;\mathrm{blood}\;\mathrm{loss};$$

When perioperative blood transfusion is performed, hidden blood loss = total blood loss + allogeneic blood transfusion + autologous blood transfusion - visible blood loss.

### Statistical analysis

All data analyses were performed using IBM SPSS 22.0 software. Categorical variables are presented as frequencies, and the chi-squared test was used to compare dichotomous variables. Normally distributed continuous variables are presented as the mean ± SD, and an independent samples t test was used to compare intergroup differences. Pearson’s correlation (used for normally distributed data), Spearman’s correlation (used for the non-normally distributed data) and multivariate linear regression analyses were performed to identify risk factors for HBL, using 13 quantitative (age, ASA classification, BMI, preoperative Hct, preoperative Cobb angle, prothrombin time, activated partial thromboplatin time, bone mineral density, operation time, segment number, pedicle screw number, autologous blood transfusion, allogeneic blood transfusion, and postoperative drainage) and qualitative variables (osteotomy, tranexamic acid and sex). For the qualitative variables, osteotomy and tranexamic acid were set as “1”. Non-osteotomy and non-tranexamic acid were set as “0”. A binary logistic regression model was established to identify the relationship between hidden blood loss and postoperative transfusion. Statistically significant continuous variables were converted to categorical variables through their cut-off points, which were determined by receiver operating characteristic curve (ROC) analysis. The level of statistical significance was set at *P* < 0.05.

## Results

### Demographic and clinical characteristics

A total of 765 patients undergoing posterior spinal fusion corrective surgeries for adolescent idiopathic scoliosis, 128 of whom were male and 637 of whom were female, were analyzed. The mean hidden blood loss volume was 693.5 ± 473.4 ml, accounting for 53.9 % of the total blood loss volume. The demographic and clinical characteristics are summarized in Table 1. The mean total blood loss volume was 1285.7 ± 437.5 ml. The mean change in Hct level was 11.0 ± 10.9 %, and the mean Hb loss was 36.8 ± 13.9 g/L. Approximately 690 (90.2 %) patients required intraoperative blood transfusions, and the mean transfusion volume was 672.4 ± 657.1 ml. A total of 205 (26.8 %) patients required transfusions of suspended red blood cells after the operation, and the mean transfusion volume was 126.5 ± 242.9 ml.

**Table 1:** Patient demographic characteristics

Parameters	Statistic
Total patients	765
Gender(M/F)	128/637
Age	14.5 ± 1.7
Weight, kg	47.9 ± 7.9
Height, cm	161.2 ± 8.0
Total blood loss, ml	1285.7 ± 437.5
Intraoperative blood loss, ml	926.8 ± 521.1
Hidden blood loss, ml	693.5 ± 473.4
Postoperative drainage, ml	464.2 ± 200.2
Change of Hct(%)	11.0 ± 10.9
Intraoperative transfusion volume, ml	672.4 ± 657.1
Postoperative transfusion volume, ml	126.5 ± 242.9
Change of Hb, g/L	36.8 ± 13.9
Mean operation time, min	250.1 ± 60.5
Mean BMI, kg/m2	18.4 ± 2.8

### Analysis of risk factors for HBL in patients undergoing scoliosis surgery

Pearson or Spearman correlation analyses for hidden blood loss showed the following parameters with *p <* 0.05 (Table 2): sex (*p* = 0.000), operation time (*p* = 0.002), preoperative Hct (*p* = 0.000), preoperative Cobb angle (*p* = 0.024), tranexamic acid (*p* = 0.000), allogeneic blood transfusion (*p* = 0.000) and segment number (*p* = 0.040). To further explore the association between HBL and the risk factors mentioned above, we performed a multivariate linear regression analysis. The results indicated that preoperative Hct (*p* = 0.003) and allogeneic blood transfusion (*p* < 0.0001) were independent risk factors for HBL, while tranexamic acid (*p* = 0.003) was negatively correlated with HBL (Table 3).

**Table 2:** Results of Pearson or Spearman correlation analyses for hidden blood loss

Parameters	Sig (2-tailed)	*P*
Age	0.102	0.058
Gender	0.137	**0.000**
BMI	-0.006	0.873
ASA	0.021	0.567
Preoperative Cobb angle	0.082	**0.024**
Preoperative Hct	0.145	**0.000**
Preoperative APTT	0.049	0.176
Preoperative PT	-0.005	0.901
Operation time	0.112	**0.002**
Bone mineral density	-0.066	0.068
Segment number	0.074	**0.040**
Pedicle screw number	0.053	0.146
Osteotomy	0.027	0.449
Autologous blood transfusion	0.009	0.803
Allogeneic blood transfusion	0.194	**0.000**
Postoperative drainage	-0.017	0.631
Tranexamic acid	-0.159	**0.000**

**Table 3:** Results of the multivariate linear regression analysis for hidden blood loss

	Unstandardized		Standardized		
coefficient	β	SE	β	t	*P* Value
Constant	-66.169	255.887		-0.259	0.796
Gender	95.745	49.444	0.075	1.936	0.053
Preoperative Cobb angle	0.792	1.502	0.019	0.528	0.598
Segment number	-15.017	7.815	-0.078	-1.922	0.055
Operation time	-0.393	0.308	-0.050	-1.274	0.203
Tranexamic acid	-126.394	41.730	-0.103	-3.029	**0.003**
Preoperative Hct	18.205	6.114	0.116	2.978	**0.003**
Allogeneic blood transfusion	0.256	0.027	0.388	9.307	**0.000**

### HBL was an independent risk factor for the requirement for postoperative transfusion in scoliosis corrective surgery patients

As illustrated in Table 4, the univariate analysis showed that variables sex, BMI, preoperative Hb, segment number, pedicle screw number, HBL and postoperative drainage were significantly different between the postoperative transfusion and postoperative nontransfusion groups. Statistically significant continuous variables were transformed into categorical variables by their cut-off points. These variables were further analyzed by binary logistic regression, and the results revealed that HBL > 850 ml (*P* < 0.001, OR: 8.845, 95 % CI: 5.806–13.290) was an independent risk factor for postoperative blood transfusion (Table 5).

**Table 4:** Univariate analysis between the postoperative transfusion group and the nontransfusion group of scoliosis corrective surgery patients

Parameters	Transfusion group	Non-Transfusion group	*P*
Age	14.3 ± 1.8	14.5 ± 1.7	0.504
Gender,male/total(%)	17/205(8.3 %)	111/560(19.8 %)	**0.000**
BMI, kg/m2	18.0 ± 3.3	18.6 ± 3.0	**0.018**
Preoperative Hb, g/L	130.3 ± 11.5	136.8 ± 11.2	**0.000**
Preoperative Cobb angle, degree	48.9 ± 12.6	47.2 ± 10.6	0.055
Preoperative Hct(%)	38.7 ± 3.0	40.3 ± 2.9	0.949
Preoperative PLT,10^9^/l	228.7 ± 52.7	232.4 ± 48.1	0.364
Preoperative INR	1.06 ± 0.07	1.05 ± 0.08	0.151
Preoperative PT, s	12.1 ± 0.9	12.0 ± 0.9	0.061
Preoperative APTT, s	32.4 ± 4.0	30.9 ± 4.0	0.983
Preoperative BUN, mmol/L	4.4 ± 1.1	4.4 ± 1.1	0.593
Preoperative SCR, µmol/L	47.2 ± 9.5	49.5 ± 10.0	0.536
ASA (I/II)	94/111	296/264	0.087
Segment number, n	10.7 ± 2.3	9.9 ± 2.5	**0.028**
Operation time, min	256.0 ± 63.3	247.9 ± 59.3	0.101
Pedicle screw number, n	15.6 ± 2.3	15.0 ± 2.7	**0.010**
Osteotomy, n(%)	30(14.6 %)	72(12.9 %)	0.549
Postoperative drainage, ml	545.3 ± 225.1	434.5 ± 181.6	**0.009**
Tranexamic acid, n(%)	22(10.7 %)	80(14.3 %)	0.230
Intraoperative blood loss, ml	997.6 ± 641.1	900.9 ± 555.9	0.057
HBL, ml	1005.9 ± 594.1	579.2 ± 358.7	**0.000**

**Table 5:** Results of binary logistic regression analysis for postoperative transfusion in scoliosis corrective surgery patients

Variables	β	SE	*P*	OR	95 %CI
Gender	1.347	0.319	**0.021**	3.845	2.056–7.192
Segment number> 11	0.569	0.221	**0.010**	1.766	1.145–2.724
BMI > 16.7 kg/m2	−0.241	0.215	0.262	0.786	0.516–1.198
Preoperative Hb> 128 g/L	−1.065	0.213	**< 0.001**	0.345	0.227–0.523
Pedicle screw number> 14	−0.189	0.243	0.436	0.828	0.514–1.333
Postoperative drainage> 425 ml	0.978	0.211	**< 0.001**	2.658	1.759–4.017
HBL > 850 ml	2.180	0.208	**< 0.001**	8.845	5.806–13.290

## Discussion

Posterior spinal fusion corrective surgeries for adolescent idiopathic scoliosis are often associated with significant intraoperative blood loss [11–13]. The volume of visible blood loss can be easily measured in the perioperative period, which can attract the attention of clinicians. In clinical practice, we always observe a degree of postoperative haemoglobin decline that is inconsistent with the volume of intraoperative blood loss. Thus, there may be other sources of blood loss. In 2000, the concept of HBL was first proposed by Sehat et al. [2], which can explain this phenomenon. However, the bleeding defined HBL which was caused by the leaking of blood into tissue spaces, blood accumulated in the surgical field and blood haemolysis is always ignored although reported with a large volume. Fortunately, an increasing number of studies on HBL after spinal surgery have been performed in recent years. Xu et al. [3] reported that the average volume of HBL after posterior lumbar interbody fusion surgery for lumbar spinal stenosis was 423 ml, accounting for 61 % of total blood loss; Derong et al. determined that HBL accounted for 47 % of the total blood loss [14]. These numbers were higher than expected. Our study showed that the mean HBL volume during scoliosis surgery was 693.5 ml, accounting for 53.9 % of the total blood loss. These results indicate that HBL accounts for a considerable proportion of perioperative blood loss; therefore, surgeons and anaesthesiologists should pay more attention to physiological derangement related to HBL.

The underlying mechanism of HBL has not been well clarified. The mainstream view is that HBL primarily occurs as extravasation of the blood into the surgical-site surrounding tissue, blood haemolysis and blood accumulated in the surgical field. Research has shown that main source of HBL is the extravasation of blood into tissues by using labelled red blood cells [15–17]. In our study, the extensive surgical area required for scoliosis surgery provided a wide area for bleeding, which consequently led to more hidden blood loss than transforaminal lumbar interbody fusion surgery [18, 19]. Therefore, paravertebral tissue incisions and wound sutures need to be more rigorously applied in corrective scoliosis surgery. Pattison et al. [20] showed that haemolysis was another possible cause of HBL. Faris et al. [21] reported that the employment of unwashed, filtered and reinfused blood increased haemolysis. Furthermore, in this study, intraoperative allogeneic blood transfusion was an independent risk factor for HBL in the multivariate linear regression analysis. Therefore, we speculated that haemolysis of erythrocytes induced by allogeneic blood transfusion might be, at least in part, responsible for HBL in posterior spinal fusion surgeries. Further prospective studies are needed to elucidate the underlying mechanism of HBL.

In this study, we investigated and examined the potential risk factors for HBL in scoliosis corrective surgery by multivariate linear regression analysis. The results showed that preoperative Hct was another independent risk factor for HBL. That is, patients with higher preoperative Hct appear to be in a state of haemoconcentration. More red blood cells are lost during intraoperative bleeding, which results in a lower Hct when combined with infusion dilution. We speculated that this may be one of the reasons for the higher HBL. Our study revealed that the use of tranexamic acid was negatively correlated with HBL. Tranexamic acid is a synthetic lysine analogue that is often used in orthopaedic surgery as an antifibrinolytic drug. Previous studies have shown that tranexamic acid can effectively reduce the total volume of intraoperative bleeding and the need for blood transfusion in scoliosis corrective surgery [22, 23], but there are few reports on whether tranexamic acid can reduce HBL in scoliosis corrective surgery. In transforaminal thoracic interbody fusion, Wang et al. found that the application of tranexamic acid significantly reduced HBL [24]. This group recommended that tranexamic acid be used as a standard drug to reduce HBL during the perioperative period. In the current study, tranexamic acid was used at a loading dose of 1 g before skin incision, and HBL was significantly reduced compared to that in the control group of posterior spinal fusion surgery patients. Therefore, intraoperative use of tranexamic acid may contribute to reducing HBL in scoliosis corrective surgery patients. However, the sample size of these studies was relatively small, and the effect of tranexamic acid on HBL, including drug dosage, still needs to be confirmed in more rigorous prospective studies with a larger sample size.

Excessive blood loss can lead to relevant postoperative complications [25], especially for minors, because their compensatory ability is less sound than that of adults. Wen et al. suggested a positive association between HBL and operative complications, although postoperative complications were not an independent risk factor for HBL [16]. The volume of HBL should be of sufficient concern to clinicians. In this research, the incidence of postoperative blood transfusion was as high as 26.8 %. Compared with intraoperative blood transfusion, postoperative anaemia is not easily detected in a timely manner. Therefore, we explored whether the volume of HBL was related to the necessity for postoperative transfusion. Binary logistic regression showed that HBL was an independent risk factor for the necessity for postoperative transfusion for scoliosis corrective surgery patients. In scoliosis corrective surgery, patients with HBL volumes greater than 850 ml, especially those with anaemia, should be closely monitored postoperatively in case of potential complications. As opposed to visible blood loss, HBL is more likely to be overlooked, especially in the postoperative period. The clinical application of hidden blood loss determination will provide clinicians with a new indicator for the management of postoperative blood transfusion, in addition to postoperative drainage volumes and haemoglobin levels, which will facilitate more accurate postoperative management strategies and ensure patients’ safety in perioperative period.

There were several limitations in this study. Since this was a descriptive study, bias may have been present. Further prospective studies are required to confirm the risk factors for HBL in scoliosis corrective surgery patients. In addition, Hct was measured twice before surgery in some patients, and the latest Hct was extracted to calculate HBL. Whether the latest measurements reflect preoperative Hct levels is still controversial. Finally, we could not rule out the effect of racial differences on HBL because most patients included in this study were native residents.

## Conclusions

In conclusion, a large volume of HBL occurred in adolescent idiopathic scoliosis patients undergoing posterior spinal fusion surgeries, and this level of HBL was higher than previously expected in posterior spinal fusion surgeries. Allogeneic blood transfusion and preoperative Hct were independent risk factors for HBL, while tranexamic acid was negatively correlated with HBL. HBL and its influencing factors should be considered when determining perioperative transfusion management strategies for scoliosis corrective surgery patients. In addition, HBL volumes greater than 850 ml were an independent risk factor for the necessity for postoperative transfusion in scoliosis corrective surgery patients. Physicians should closely monitor patients with hidden blood loss volumes greater than 850 ml in cases of postoperative anaemia.

## Data Availability

The datasets generated during and analyzed during the current study are not publicly available due to hospital regulations but are available from the corresponding author on reasonable request.

## Abbreviations

HBL: Hidden blood loss
Hct: Hematocrit
Hb: Hemoglobin
BMI: Body mass index
ASA: American society of Anesthesiologists
PLT: Platelet
INR: International Normalized Ratio
APTT: Activated partial thromboplastin time
PT: Prothrombin time
BUN: Blood urea nitrogen
SCR: Serum creatinine
